# Development and Effectiveness Verification of Metaverse Cognitive Therapy Contents for MCI Patients

**DOI:** 10.3390/s23136010

**Published:** 2023-06-28

**Authors:** Gi Sung Oh, Jehyun Kim, Wonjun Jeong, Seokhee Oh, Taeg Keun Whangbo

**Affiliations:** 1 Department of IT Convergence Engineering, Gachon University, Seongnam-si 13120, Republic of Korea; 2 Korea Creative Content Agency, Daejeon Metropolitan City 34863, Republic of Korea; 3 Department of Computer Engineering, Gachon University, Seongnam-si 13120, Republic of Korea

**Keywords:** metaverse, VR, MCI, cognitive therapy, mixed multimedia contents

## Abstract

It is very important to prevent dementia by intervening in advance in the stage of mild cognitive impairment, which is the pre-stage of dementia. Recently, cognitive therapy research using metaverse has been on the rise. We propose a way to utilize metaverse cognitive therapy content as a non-drug treatment method of mild cognitive impairment patients. This paper shows the results of clinical trials using metaverse cognitive therapy contents developed by us. We collected data from MCI patient groups and normal groups through MMSE-KC tests and in-content data collection systems. We conducted paired *t*-tests and repeat measurement ANOVA based on the collected data. The results of this study show how metaverse cognitive therapy content affects MCI patients, and suggest various factors to be considered when creating functional content.

## 1. Introduction

According to a WHO (World Health Organization) report, there are currently more than 55 million patients with dementia worldwide, and it is expected to increase to 78 million in 2030 and 139 million in 2050 [1]. Dementia means a state of being unable to live a daily life on one’s own due to cognitive impairment [2]. It has a great impact not only on the lives of patients but also on their families and society as a whole.

Now that our aging society is accelerating due to extended life expectancy and reduced fertility rates, dementia is becoming one of the major social problems in major developed countries. Dementia can be subdivided into about 70 different types depending on the cause, of which Alzheimer’s dementia accounts for the majority [3]. Alzheimer’s dementia mainly causes cognitive problems due to decreased brain function caused by physical aging [4]. There are numerous causes, but in common it is confirmed that cognitive function decreases due to the degeneration of brain cells. Therefore, the main age group of dementia patients is in their mid-70s to early 80s. However, since there is not necessarily a causal relationship with physical aging, dementia can occur even at a relatively young age [5].

Drug therapy is used in the therapy of dementia, and choline esterase inhibitors are mainly used to inhibit the breakdown of acetylcholine, a neurotransmitter necessary to maintain cognitive functions, including memory, to prevent worsening symptoms of dementia [6]. However, it slows the progression of dementia and is not a complete therapy.

As there is still no complete cure for dementia, it is very important to prevent it in advance [7]. Patients with early symptoms of cognitive impairment can effectively delay the progression to dementia via lifestyle changes and cognitive training [8,9]. Therefore, we should pay attention to the stage of cognitive decline, which is the stage before it progresses to dementia. Cognitive decline can be confirmed through interviews between patients and guardians, and cognitive function tests (CERAD, MMSE, etc.) [10]. The stages of cognitive decline are classified into subjective cognitive impairment and mild cognitive impairment, the latter of which are clinically okay but subjectively complain of forgetfulness or personality changes [11].

Various methods are being used to treat MCI. MCI therapy is divided into drug therapy using Alzheimer’s therapy and antidepressants, and cognitive rehabilitation non-drug therapy for brain function in the degraded area [12]. In the case of drug therapy for MCI patients, it is reported that drugs struggle to obtain many therapeutic effects and have no long-term therapeutic effects. In addition, since many older people already take a lot of drugs from existing diseases, the importance of non-drug therapy is attracting attention as the risk of drug interaction is high [13]. Additionally, therapy with pharmacological therapy alone is limited, and when combined with two types of therapy, the therapeutic effect increases.

Non-drug cognitive therapy methods include cognitive stimulation, cognitive training, and cognitive rehabilitation [14]. First of all, cognitive stimulation is a precautionary action to prevent cognitive decline in normal people. Cognitive training trains in an way integrated different areas, such as memory, attention, execution, and communication. On the other hand, cognitive rehabilitation refers to a program to improve one specific cognitive function that has been degraded. In order to improve the cognitive function, the task for improvement is intensively carried out in a set way. Among them, therapy for MCI patients uses a combination of cognitive training and cognitive rehabilitation methods.

Cognitive training and cognitive rehabilitation create programs based mainly on daily activities, time, and places to improve memory, thinking, and attention, and use them to treat them [14]. Watching watches, shopping, and doing laundry utilize certain patterns and association memories to improve adaptability and judgment. This is effective in promoting the overall improvement of complex cognitive and social functions. However, there are some shortcomings. In most cases, it is okay to proceed in a one-to-many format, but certain training requires intensive help from a doctor or therapist in a one-on-one form. There are also restrictions on time and space. Patients need to go to the hospital for therapy, and it is also necessary to utilize a dedicated therapy space for training in certain scenarios. Due to this hassle, even if they receive therapy through a doctor’s surgery, they sometimes give up therapy in the middle.

Recently, in order to compensate for these shortcomings, various attempts have been made towards providing realistic therapy content or counseling space using the virtual world [15,16]. The cognitive therapy method using the metaverse environment presents a clear direction of therapy and motivates patients [17,18]. The virtual environment can be used to provide a situation that enables dynamic testing and training without time and space constraints. Many researchers are using metaverse environments for various purposes based on this point, and more and more cases have been shown in cognitive therapy research [19,20,21].

The metaverse refers to various forms or contents that implement real-life interactions in a virtual space. This includes all areas where reality and the virtual space interact, such as virtual reality, augmented reality, mixed reality, and augmented reality [22]. However, it has unique characteristics that are different from existing concepts. It is the extension of everyday life and the connection between users. The metaverse can incorporate daily life into a different worldview, and create another immersive society through interaction and sharing between users.

Cognitive therapy using the metaverse creates a virtual artificial environment based on scenarios. It provides patients with an immersive therapy environment by wearing a wearable device, an HMD (head-mounted display), and allows them to interact with virtual objects. This strengthens patients’ motivation for participation based on fun, allowing users to make impossible choices in other ways and create positive changes [23]. MCI patients can perform repetitive exercises according to guidelines given in a virtual environment to continuously remember information and improve concentration. The realism and presence in virtual space improves the patient’s spatial perception in a more positive direction than traditional PC- and textbook-based counseling therapy [24]. Metaverse content also provides a window for patients to communicate with other users, and promotes dialogue with new people. This helps socially isolated patients improve their overall quality of life and improve their symptoms [25].

Research is increasing to prove the effectiveness of cognitive therapy using the metaverse in various areas of mental illness as well as MCI. Some have found that therapies using virtual environments are less ethically problematic than actual field-based therapies [23]. It also provides scientific justification for the medical use of metaverse content based on objective bio-signal data, such as EEG, and statistical evidence using cognitive measurement tools such as neuropsychological evaluations [24].

However, cognitive therapy for MCI patients using metaverse environments is still limited, and more samples are needed for research cases. Since MCI patients are mostly older people in their 70s and 80s, long-term use for them is difficult, and physical discomfort such as nausea, dizziness, cyber sickness, and eye fatigue can occur during long-term use [26,27]. Additionally, if one looks at existing research cases, one can see some UX/UI and low-quality content that does not take into account the user, a content scenario with insufficient medical verification, and an unstructured experimental environment. Therefore, this study developed UX considering patients who can be used for cognitive therapy purposes and high-quality content that can increase the effect through continuous immersion and fun.

Using this, this paper aims to verify the clinical effect of this content as a cognitive therapy tool by applying a developed program for MCI patients. In the research process, we produced content based on cognitive therapy scenarios confirmed by psychiatrists, and conducted clinical trials by recruiting university hospitals and clinical subjects. In addition, cognitive measurement tools that are traditionally used were used to confirm any changes in cognition. Currently, there are few cases of quantitative clinical verifications of using metaverse cognitive therapy content for MCI patients in domestic research cases, so this study can be used as one case. In addition, it can be expected that the actual medical effect is meaningful, and we can check the content production process and clinical process for older patients.

## 2. Development of Metaverse Cognitive Therapy Contents for MCI Patients

This stage deals with the process of producing metaverse content for cognitive therapy purposes for MCI patients. We have developed a variety of MCI response scenarios that have been confirmed by psychiatrists and clinical trial specialists. Based on this, we have developed contents and repeated modifications according to the confirmations by medical staff during the development process.

### 2.1. Content Scenario Development

We designed a metaverse content scenario for cognitive therapy purposes in MCI patients. The scenario is designed to train around memory, attention, and execution functions, and the development process refers to a number of traditional MCI cognitive therapy methods. Content experts, psychiatrists, and clinical trial experts participated in the production of the scenario. As a result, the scenario shown in Table 1 below was produced.

There are five scenarios designed: shopping, cooking, banking, visiting a hospital, and taking a walk. It is composed of scenarios using a corrective approach centered on daily activities. All scenarios include gamification components and allow the active use of space with random conditions for interaction.

### 2.2. Development of Cognitive Therapy Contents

**Figure 1:**
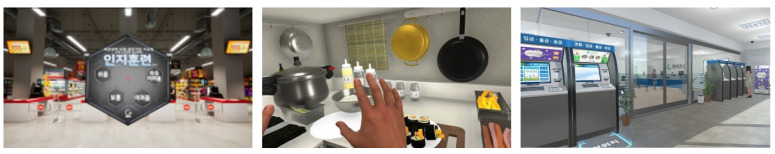
Developed VR cognitive training content.

We used Unreal Engine 4 to produce Metaverse contents. Unreal Engine 4 is an integrated game engine developed by Epic Games in the U.S. that supports multiple platforms and features high-quality real-time live action graphics expression. It is used for commercial game production and the production of metaverse, VR, and AR contents. This content was produced in a form optimized for Samsung Odyssey VR HMD based on Unreal Engine 4. The following Figure 1 is a scene showing some of the content produced.

**Figure 2:**
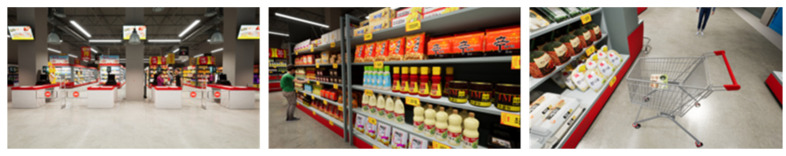
Product purchase content within the developed VR cognitive training content.

The metaverse cognitive therapy content used in our experiments is shopping content. This content is based on a real market. We subdivided the space into seven product categories, including dairy products, processed foods, and meat, and organized them to be interacted with by placing appropriate objects that can be purchased in each category. The condition for content performance is to remember the purchase list shown at the entrance of the market, put the right product in the shopping basket, and arrive at the checkout counter. Users can choose a level of difficulty from 1 to 4. The number of items to be purchased at difficulty level 1 is four, and each time the difficulty level increases, the number of items to remember increases by one. During the experience, subjects can use hints to recheck the purchase list. Figure 2 shows more of the shopping content.

## 3. Experiment Process and Method

### 3.1. Research Procedure

**Figure 3:**
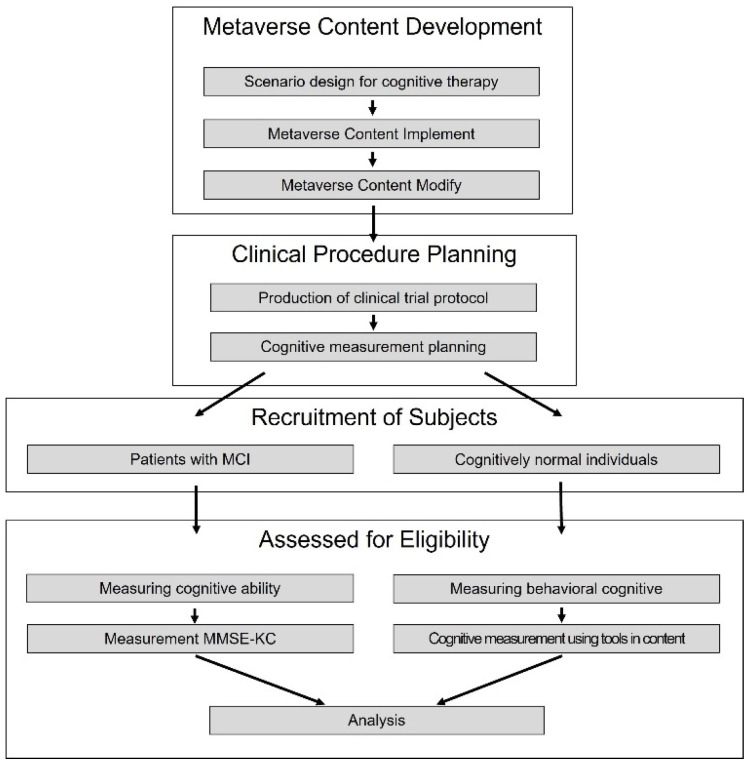
Research procedure.

We did our research in accordance with the procedure as shown in Figure 3. First, we went through the content scenario production process of the previous chapter, and based on this, we developed cognitive therapy content. After that, we planned a clinical trial procedure using the content. First of all, in cooperation with Gachon University Gil Hospital, Samsung Seoul Hospital, and Inha University Hospital, MCI patients and normal people who were admitted to each hospital were recruited as subjects. In addition, research consent was sought from the recruitment target and eight clinical trials were conducted. Cognitive measurements using cognitive measurement tools were conducted at the beginning and end of the sessions to collect data.

### 3.2. Recruit Subjects

We recruited MCI patients and normal subjects to participate in this clinical trial through each hospital’s research website. A total of 56 participants were recruited for this experiment, consisting of 31 in the MCI group and 25 in the cognitive group. Originally, it was planned to set up an MCI control group to check the differences between the same MCI subjects, but in order to form a subject group of at least 30 people each, the composition of the MCI control group was abandoned.

Since MCI is mainly expressed in older people in their 70s and 80s or older, the recruitment target of this experiment was also limited to those in their 70s and older. The MCI criteria diagnosis of subjects included in the MCI group was based on the following criteria (Peterson, et al., 1999 [28]):

(1) Appeal for memory loss by a patient or guardian;
(2) Normal daily life;
(3) In the cognitive intelligence test, considering the age and education level, memory damage below −1.5 standard deviation compared to the average is identified.

In addition, those who met the same criteria as Table 2 were excluded from both MCI and normal subject groups. Table 3 represents the general characteristics of the subjects involved in this clinical trial.

### 3.3. Compliance with Research Ethics

In order to comply with the safety and bioethics of the subjects participating in the study, this study implemented a procedure for consent to participate in the study based on the following steps. First, we explained the purpose of the study to both the MCI subjects and normal subjects involved in the study, and informed them that the data collected were to be used solely for research purposes. They were told that if they felt burdened by participating in the study or if questions were raised about the research process, they could stop participating at their discretion. In this research process, the safety of participants comes first, and it was guaranteed that there were no side effects or risks from participating in the study. Finally, the criteria for exclusion in each clinical trial, as shown in Table 4, were notified. All research participants were informed of the above information about the study, and we obtained written consent from each participant. Finally, this study was approved by the Review Committee on Clinical Research Ethics at Gachon University Gil Hospital on 13 February 2018 (IRB: GAIRB2018-052).

### 3.4. Clinical Trial Conducted

Clinical trials were conducted from June 2018 to December 2019 at Gachon University Gil Hospital, Samsung Medical Center, and Inha University Hospital. For the smooth performance of the clinical trials, all operations were carried out under the supervision of a clinical therapist. After the first and eighth sessions, cognitive measurements of the subjects were performed with a cognitive measurement tool. They were trained on this program and how to use VR HMD before performing the cognitive program. The HMD used in the clinical trials was a Samsung HMD Odyssey. All subjects performed the cognitive therapy programs twice a week for four weeks, for a total of eight sessions. If there as any health problem with the subject during the program, rest and medical checkups were prescribed with immediate termination. The cognitive therapy program was performed in a sitting or standing position according to the subject’s preference.

### 3.5. Measurement Tools and Data Analysis

The measurement tools used during this clinical trial included MMSE-KC and data designed for the content. The MMSE-KC (Mini-Mental Statue Examination) is a simple psychiatric test made by translating the MMSE, which is included in the English version of the CERAD (Consortium to Establish a Registry for Alzheimer’s Disease) evaluation book, to suit Koreans. The MMSE-KC has standards for older people in Korea and has standardized questions so that they are relatively less affected by their academic background [29]. Therefore, it is widely used to measure cognition and to test for dementia in a simple form.

The data measurement system here was designed to measure the number of times they found items in the market, the number of times they put the wrong items in the cart, and the number of times they used hints in each session, then record them in log files, and send them to the DB. When there was a significant change in the results using previously used cognitive measurement tools, we verified the effectiveness of this content by checking whether significant changes occurred in the data collection results.

The statistical analysis of all data was done using SPSS (Version 25, SPSS Inc., Chicago, IL, USA). First, the Shapiro–Wilk normality test was conducted, and a paired *t*-test was conducted to confirm changes in each group before and after this program was carried out. Afterwards, the changes in score of the MCI patient group and the normal group before and after cognitive therapy were confirmed using the repetitive measurement ANOVA.

## 4. Experimental Results

### 4.1. MMSE-KC Score Result Analysis

The total score of the MMSE-KC is 30 points; more than 24 points is normal, 20 to 23 points is suspected of dementia, and 19 points or less is judged to be definitive dementia. Therefore, in general, a score of between 23 and 24 points is used as a measure of cognitive impairment.

Of the total 56 subjects, 11 were left out, 9 from the MCI group and 2 from the normal group. Therefore, based on 45 people, the data were analyzed with 22 people in the MCI group and 23 people in the normal group. First, we derived the paired *t*-test results for each subject group’s MMSE-KC total score. The significance level was set at 0.05. Table 4 shows the result.

We saw significant changes between before and after the program, based on the entire group of subjects. First, we could see that the average of total points increased before and after the session (24.38 ± 4.08 vs. 25.29 ± 3.57, *p* < 0.05). The change in the total score was also significant in the MCI group, indicating that the score changed to a level close to the normal standard after the program (21.68 ± 3.62 vs. 23.73 ± 3.72, *p* < 0.05). However, in the normal group, it was confirmed that the total score itself increased from before to after, but there was no significant difference. Through this, we are able to confirm that the cognitive therapy programs were effective for the MCI group.

### 4.2. Data Statistics in Contents

The data that can be collected while performing the program are as follows:

(1) Total performance time of contents;
(2) Number of hints used;
(3) The number of items picked up;
(4) The number of correct answers (the number of times they put the right item in the cart);
(5) The number of incorrect answers (the number of times they put the wrong item in the cart).

Factors (1), (2), (3), and (5) are negative, as the numbers decreased from before to after, and (4) is a positive indicator, as the number increases. We compared before and after based on the data obtained by performing Session 1 and Session 8 four times each.

#### 4.2.1. Change in Content Total Performance Time

We conducted a repetitive measurement ANOVA to verify the change in the total performance time of the content by each subject group. Table 5 shows the results.

In the MCI patient group, the average total duration of the session decreased before and after the session, which was a significant change (230.78 ± 42.68 vs. 207.50 ± 20.02, *p* < 0.05). In the normal group, the average value of the total performance time instead increased between before and after the session, which was not a significant change.

#### 4.2.2. Change in the Number of Hints Used

We conducted a repetitive measurement ANOVA to verify the change in the number of hints used by each subject group. Table 6 shows the results.

In the MCI patient group, the average value of the total number of hints used decreased between before and after the session, which was a significant change (1.79 ± 0.59 vs. 1.11 ± 0.29, *p* < 0.05). In the normal group, we also found that the average number of hints used decreased between before and after the session, which was a significant change (1 ± 0.34 vs. 0.6 ± 0.16, *p* < 0.05).

#### 4.2.3. Change in the Number of Items Picked Up

We conducted a repetitive measurement ANOVA to verify the change in the number of items picked up by the subject group. Table 7 shows the results.

In the MCI patient group, the average value of the total number of items picked up increased between before and after the session, but it was confirmed that this was an insignificant change. In the normal group as well, the average value increased between before and after the session, confirming that this was a significant change (5.18 ± 0.15 vs. 11.68 ± 1.82, *p* < 0.05).

#### 4.2.4. The Number of Correct Answers (the Number of Times They Put the Right Item in the Cart)

We collected data on the numbers of correct answers for all subjects, but the number of correct answers for all subjects equally increased from four to seven. It is confirmed that this was because a perfect score at the lowest level of difficulty is four points in the first session, and a perfect score in the eighth session is seven points. The reason was that the session was set to end only when the correct item was inserted in the cart. Therefore, it was a content design problem, which made it impossible to use the score statistically.

#### 4.2.5. The Number of Incorrect Answers (the Number of Incorrect Items Put in the Cart)

We confirmed that the number of incorrect answers, unlike the number of correct answers, had nothing to do with the termination of the content. Therefore, it was determined that this figure could be used statistically. We conducted a repetitive measurement ANOVA to verify the change in the number of incorrect answers for each subject group. Table 8 shows the results.

In the MCI patient group, the average number of incorrect answers increased between before and after the session, which was not a significant change. In the normal group, we found that the average number of incorrect answers increased between before and after the session, but this was a significant change (0.68 ± 0.38 vs. 4.03 ± 1.04, *p* < 0.05).

#### 4.2.6. Discussion

We compared the differences between before and after the program under five conditions. Statistical analysis was possible in the rest, except for the number of correct answers. Among these, the conditions that showed significant changes in the MCI patient group were the total performance time of the content and the number of hints used. In the normal group, significant changes were found in the number of hints used, the number of items collected, and the number of incorrect answers. The variables that showed the amount of change in the figures in advance were the total amount of time spent on content and the number of hints used.

First we paid attention to the total execution time of the content. We were able to see the figures decreasing over time, as we thought. Given that the before-to-after average error in the normal group is not large, and the standard deviation is also low in the normal group, it is estimated that the figure could no longer be lowered due to the low average performance time. Therefore, based on these changes in a group of MCI patients in an impaired cognitive state with a low ability to adapt to an unfamiliar environment, this result can be taken as proving the effectiveness of the content.

Next, we could see that the use of hints by both subject groups decreased in eight sessions compared to in one session, both of which were statistically significant. First of all, the same number of hints was used regardless of the difficulty of the content, depending on the subjects’ needs. Additionally, all purchase list conditions are randomly constructed according to random numbers. Therefore, we found that the number of hints used was affected by each subject’s memory, which is considered as proving the effectiveness of the content.

In addition, we concluded that the number of items picked up, the number of correct answers, and the number of incorrect answers do not appear to prove the effectiveness of the content. First of all, in the case of the number of items picked up, there are a number of cases in which the item can be held without intention, or can be held incorrectly, depending on the user’s proficiency in using the HMD controller. The number of correct answers is as described in the previous statistics, and in the case of the number of incorrect answers, there is also a possibility that they were put in the wrong cart depending on the proficiency of using the HMD controller. Through this result, we were able to confirm that when designing functional content, we should consider the figures that change regardless of the user’s proficiency or intention.

In this regard, we judged that metaverse contents are effective in improving the symptoms of MCI patients, and in order to increase future effectiveness, it is necessary to adjust performance time and memory learning patterns by adjusting the difficulty and complexity of contents.

Our study had a sample count of 45, and it may be difficult to say that we have a sufficiently large number of samples to generalize the results. In addition, the short four-week clinical trial for each subject is regrettable. Due to the nature of mental illness, long-term observational research is required, so follow-up research needs to be carried out in consideration of these limitations.

However, this study is highly appreciated, in that it is based on the results of practical clinical trials amongst a number of subjects. In fact, there are various difficulties in recruiting MCI patients, such as refusal and health problems. Therefore, at this point in time, this study is deemed worthwhile.

## 5. Conclusions

Through this study, we checked whether metaverse cognitive therapy content can alleviate MCI symptoms. To this end, we produced a metaverse cognitive therapy program, recruited a group of subjects, and conducted clinical trials.

The changes before and after cognitive therapy in the MCI patient group and the normal group are as follows. First, after the program, the average MMSE-KC total score of the MCI group increased. Second, the total amount of time spent on content and the number of hints used in the MCI patient group were lowered between before and after treatment. This result seems to be the result of improved memory and judgment among the cognitive abilities of the MCI patient group.

To sum up, the metaverse content developed in this study can be used in the non-drug treatment process to relieve symptoms in MCI patients. This suggests that other mental disorders can also utilize metaverse content as an alternative method to using drugs for treatment. This study proves the possibility of developing Digital Therapeutics, which has recently attracted attention, and we plan to conduct active research in this field in the future. In addition, systemic elements of the developed content and cognitive measurement tools used in clinical trials can be referred to for the development and verification of other functional content.

## Figures and Tables

**Table 1:** List of produced scenarios.

Scenario Name	Contents	Effect
Shopping	Product selection according to the purchase list, cart included	Memory, judgment
Cooking	Choosing ingredients, cooking in order	Attention, execution ability
Banking	Deposit, withdrawal, remittance	Memory, attention
Visiting a hospital	Reception to the hospital, visiting the department wanted	Execution ability, attention
Taking a walk	Communicating with people, sitting on a bench	Communication, judgment

**Table 2:** MCI and normal subject exclusion criteria.

Exclusion Criteria
Individuals with communication difficulties Individuals with cardiovascular disease Individuals with serious physical conditions that may affect cognition Individuals with severe mental conditions that may affect cognition Individuals with severe vertigo Individuals with acute psychotic symptoms Individuals with major neurocognitive disorders Individuals with severe psychiatric symptoms (CGI-S score ≥ 5)

**Table 3:** General characteristics of the subjects.

	MCI Patients	Normal Group
Age, mean (SD)	74.23 (7.50)	71.45 (3.95)
Sex (man), *n* (%)	8 (25.8)	3 (12)
Sex (women), *n* (%)	23 (74.2)	22 (88)
MMSE-KC, mean (SD)	21.68 (3.62)	26.22 (2.57)
School years completed, mean (SD)	8.68 (4.61)	8.91 (3.16)

**Table 4:** Paired *t*-test results for MMSE-KC total score by subject group.

Group	Before Training (Session 1)	After Training (Session 8)	*t* (z)	*p*
Total	24.38 ± 4.08	25.29 ± 3.57	−2.59	0.00 *
MCI patients	21.68 ± 3.62	23.73 ± 3.72	−4.06	0.00 *
Normal group	26.22 ± 2.57	27.78 ± 2.73	0.46	0.64

* *p* < 0.05.

**Table 5:** Repeated measures ANOVA results of total execution time of contents.

Group	Mean ± Std. Dev.		*F*	*p-Value*	*F Crit*
	Before Training (Session 1)	After Training (Session 8)			
MCI patients	230.78 ± 42.68	207.50 ± 20.02	23.16	0.00 **	4.75
Normal group	151.45 ± 20.33	162.43 ± 6.08	0.23	0.64	4.75

** *p* < 0.05.

**Table 6:** Repeated measures ANOVA results of the number of hints used.

Group	Mean ± Std. Dev.		*F*	*p-Value*	*F Crit*
	Before Training (Session 1)	After Training (Session 8)			
MCI patients	1.79 ± 0.59	1.11 ± 0.29	11.72	0.01 **	4.75
Normal group	1 ± 0.34	0.6 ± 0.16	8.16	0.01 **	4.75

** *p* < 0.05.

**Table 7:** Repeated measures ANOVA results of the number of items picked up.

Group	Mean ± Std. Dev.		*F*	*p-Value*	*F Crit*
	Before Training (Session 1)	After Training (Session 8)			
MCI patients	6.5 ± 0.52	11.02 ± 1.97	0.24	0.63	4.75
Normal group	5.18 ± 0.15	11.68 ± 1.82	64.84	0.00 **	4.75

** *p* < 0.05.

**Table 8:** Repeated measures ANOVA results of the number of incorrect answers.

Group	Mean ± Std. Dev.		*F*	*p-Value*	*F Crit*
	Before Training (Session 1)	After Training (Session 8)			
MCI patients	0.89 ± 0.29	3.07 ± 1.34	0.70	0.41	4.75
Normal group	0.68 ± 0.38	4.03 ± 1.04	39.51	0.00 **	4.75

** *p* < 0.05.

## Data Availability

Not applicable.
